# Knapsack - TOPSIS Technique for Vertical Handover in Heterogeneous Wireless Network

**DOI:** 10.1371/journal.pone.0134232

**Published:** 2015-08-03

**Authors:** E. M. Malathy, Vijayalakshmi Muthuswamy

**Affiliations:** Department of Information Science and Technology, College of Engineering, Anna University, Chennai, India; Semmelweis University, HUNGARY

## Abstract

In a heterogeneous wireless network, handover techniques are designed to facilitate anywhere/anytime service continuity for mobile users. Consistent best-possible access to a network with widely varying network characteristics requires seamless mobility management techniques. Hence, the vertical handover process imposes important technical challenges. Handover decisions are triggered for continuous connectivity of mobile terminals. However, bad network selection and overload conditions in the chosen network can cause fallout in the form of handover failure. In order to maintain the required Quality of Service during the handover process, decision algorithms should incorporate intelligent techniques. In this paper, a new and efficient vertical handover mechanism is implemented using a dynamic programming method from the operation research discipline. This dynamic programming approach, which is integrated with the Technique to Order Preference by Similarity to Ideal Solution (TOPSIS) method, provides the mobile user with the best handover decisions. Moreover, in this proposed handover algorithm a deterministic approach which divides the network into zones is incorporated into the network server in order to derive an optimal solution. The study revealed that this method is found to achieve better performance and QoS support to users and greatly reduce the handover failures when compared to the traditional TOPSIS method. The decision arrived at the zone gateway using this operational research analytical method (known as the dynamic programming knapsack approach together with Technique to Order Preference by Similarity to Ideal Solution) yields remarkably better results in terms of the network performance measures such as throughput and delay.

## Introduction

Next-generation heterogeneous networks support an interoperability environment that demands seamless mobility management. To enable ongoing communication activity, handover technology is designed to allow users to roam across networks. When the connection switches between different networks, the handover is known as vertical handoff, whereas handoff that takes place within the same network is referred to as horizontal handoff. The vertical handover process involves three phases [1], i.e., the system discovery phase, handoff decision phase and handoff execution phase. Providing efficient association with a suitable wireless network remains an open challenge in heterogeneous networks. Recent research has suggested few approaches that give directional assistance in selecting a candidate network during the handoff decision phase. However, it has become essential to offer an efficient handover mechanism to meet different QoS requirements and maximize the resource utilization of the network. In addition, the literature reveals that various metrics such as signal strength, bandwidth, bit error rate, power consumption, and cost evaluation to determine the selection of a candidate network. These metrics might be suitable when horizontal handoffs are initiated but become insufficient to trigger vertical handoffs due to different system characteristics. Design of the required Quality of Services (QoS)-aware vertical handover decision algorithm is another major concern in such a scenario. The vertical handoff mechanisms are generally grouped in terms of mobile-controlled handoff and network-controlled handoff processes. Various mobility management strategies for QoS support designed in the literature are based on mobile-controlled handoff. Therefore, such a design cannot select the best network for mobile devices because the device has limited power and limited information on the networks. In such a case, the ping-pong effect is high because the selection of the network is based on previous history stored in the mobile terminal. System efficient performance in terms of handover delay and the handover decision degrades due to this handover failure. The methods reported in the literature so far to solve this complex problem suffers from one or more of the following limitations—inappropriate vertical handover routing metrics, lack of proactive congestion check in the candidate network and mobile-controlled handoff that are addressed in this study. The goals of the proposed work are to reduce handover failures and to derive the required QoS support based on network controlled handoff for heterogeneous networks. The first stage of the proposed work implements the Technique to order preference by similarity to the ideal solution (TOPSIS) method, which is defined under the Multi-Attributed Decision Making method (MADM). Using this method, the appropriate ranking for each decision variable involved in the network model is obtained. The second stage of this work carries the recursive dynamic programming construction with zone-based decisions imposed on the network control side. This paper presents a new and efficient dynamic programming approach known as TOPSIS with Knapsack Vertical handover Decision (TOPSIS-KVD), an optimal solution for vertical handover challenges in next-generation networks with greatly reduced handover failures. The limit for the mobile terminal in performing handover with network congestion under check is decided via dynamic programming that determines the decision alternatives for the best network selection. The proposed TOPSIS KVD generates vertical handover decisions by taking into consideration of the significant metrics such as signal strength, RTT, reliability, cost and traffic handling priority of the candidate network. This work demonstrates an efficient direction for optimal network selection, thereby reducing the challenges of handover failure between WiMax networks and Wi-Fi networks for various QoS traffic classes. The proposed TOPSIS-KVD incorporates a simple design that achieves seamless handover over fuzzy based design and removes the ping-pong effect.

The remainder of this paper is organized as follows. Section 2 provides an overview of selected related works for vertical handover decision mechanisms. Section 3 presents a system model for implementing the proposed TOPSIS-KVD approach for the selection of the optimal network, and the simulation results are presented in Section 4. Conclusion and observations are provided in Section 5. Section 6 gives the current limitations of the proposed work and future direction.

## Related Work

A comparative study of different vertical handover algorithms is provided by Kumar [2]. The literature reveals that parameters such as signal strength, bandwidth, and power consumption influence the network characteristics that have direct impact on the design of the handover decision algorithm. Therefore, selection of parameters for the decision phase is critical for the performance evaluation of the algorithm. The Analytic Hierarchy Process (AHP) introduced by Saaty [3] is a decision support tool that uses a multi-level hierarchical structure of objectives, criteria and alternatives to solve complex decision problems. Multi-Attribute Decision Making methods (MADM) motivated Song et al [4] to address such issues in the vertical handover algorithm and to present a handover mechanism between UMTS and WLAN networks. A case study by Bhosale et al [5] revealed that decision parameters affect the approximate comparisons with priorities for WLAN, WiMax and UMTS network alternatives for simpler implementation. In addition, Savitha [6] presented a handoff decision based on a MAMD approach. However, QoS support is not designed and increases the QoS provision for the user; thus, this situation demands an additional QoS handover decision algorithm. Ghahfarokh et al [7] proposed a context-aware handover mechanism that provides better performance for delay-sensitive applications together with a MAMD method. Maaloul et al [8] improved the QoS in a heterogeneous network with user perceived satisfaction. A study by Jakimoski [9] revealed that vertical handover is performed based on mobile velocity. Although the implementation complexity is low in such findings, the system performance is not addressed when the mobile terminal moves away from the point of attachment. Hence, the mobile terminal does not identify the best solution in such a scenario. Policy-based QoS support presented by authors Stevens [10] and Singhrova et al [11] allows policy adjustment by the network operator to meet the required QoS in a dynamic environment. However, it becomes essential to supply QoS for various traffic classes. Addressing the required QoS becomes an imperative factor for heterogeneous networks. Bellavista et al [12] addressed the crucial problems of service continuity during vertical handoffs, and Kantubukta et al [13] proposed an application-specific handoff method based on the fuzzy logic concept. Fuzzy logic and neural network-based decision algorithms provide high throughput and low blocking probability for the decision procedure. The literature [14][15]reveals that an energy efficient handover mechanism with QoS guarantee is designed with mobile-initiated handover. However, the delay in handover process still exists. Also it can be seen that a mobile device is too small to handle a large set of handover decisions demanding highly sophisticated memory management techniques. Adnan et al [16] presented a survey of comprehensive works performed on a QoS-aware vertical handover decision algorithm. The paper presented a compressive survey on vertical handover algorithms and assessment gave clear view about the complexity on implementation of these algorithms. The reveals that lack of network parameter greatly influence the performance of vertical handover process. Also literature by Khattab [17] presented a survey on MIH(Media Independent Handover and ANDSF(Access Network Discovery and Selection Function) schemes for vertical handover. ANDSF protocol proposed in literature [18] works with RAT information to perform handover decision. This scheme is of medium complexity and new logical entities were added to provide authentication and link connection during handover. This mechanism enables seamless handover with set of rules for traffic routing. Hence the validity of these rules are periodically re-evaluated by the device which in turn adds an additional power management challenge to the mobile device. Also Hotspot 2.0 which is based on the IEEE 802.11u standard are used in literature [19] to support vertical handover. Hotspot 2.0 Management Object degrades selection process for candidate network on dynamic conditions. This is due to the unavailability of information specific to traffic condition. Also conflicting rules raises due to inconsistent sets of information from different sources that leads to complex information management which needs to be addressed. Moreover, the intelligent schemes developed should be flexible and expandable to include additional QoS parameters of interest to vertical handover decisions. This motivates the design of seamless vertical handover approach.

Moreover, it can be clearly understood from the literature survey that there is no evidence in the past which employed dynamic programming with the Knapsack problem approach to handle various QoS traffic classes for vertical handover problem. The proposed handoff decision strategy is designed for network-controlled handoff, which erratically removes the limitation of mobile-controlled handoff. This situation motivates the division of the network into zones, and computation of the ranking score by TOPSIS method offers a deviation of the decision variable towards the ideal solution. The proposed schemes adds repository to keep all set of policies governing the decision rules and network makes smart choice to choose the candidate network through MADM method such as TOPSIS. Moreover, TOPSIS alone is unreliable for performing handover in a heterogeneous environment. To adopt traffic load balance in determination of optimal VHO triggers, dynamic programming steers the correct mobile user with optimal handover through mobile IP functionality. This paper adds QoS-aware decisions to improve efficiency by reducing unnecessary handover and removing centralized estimation of the network via estimation of the decision using a dynamic approach in the handover scheme. To achieve an optimal solution, recursive dynamic programming construction is derived, which uses stage-wise analysis of the mobile nodes connected to the point of attachment/base stations.

## System Model

### 3.1 Architecture of Proposed System

**Fig 1:**
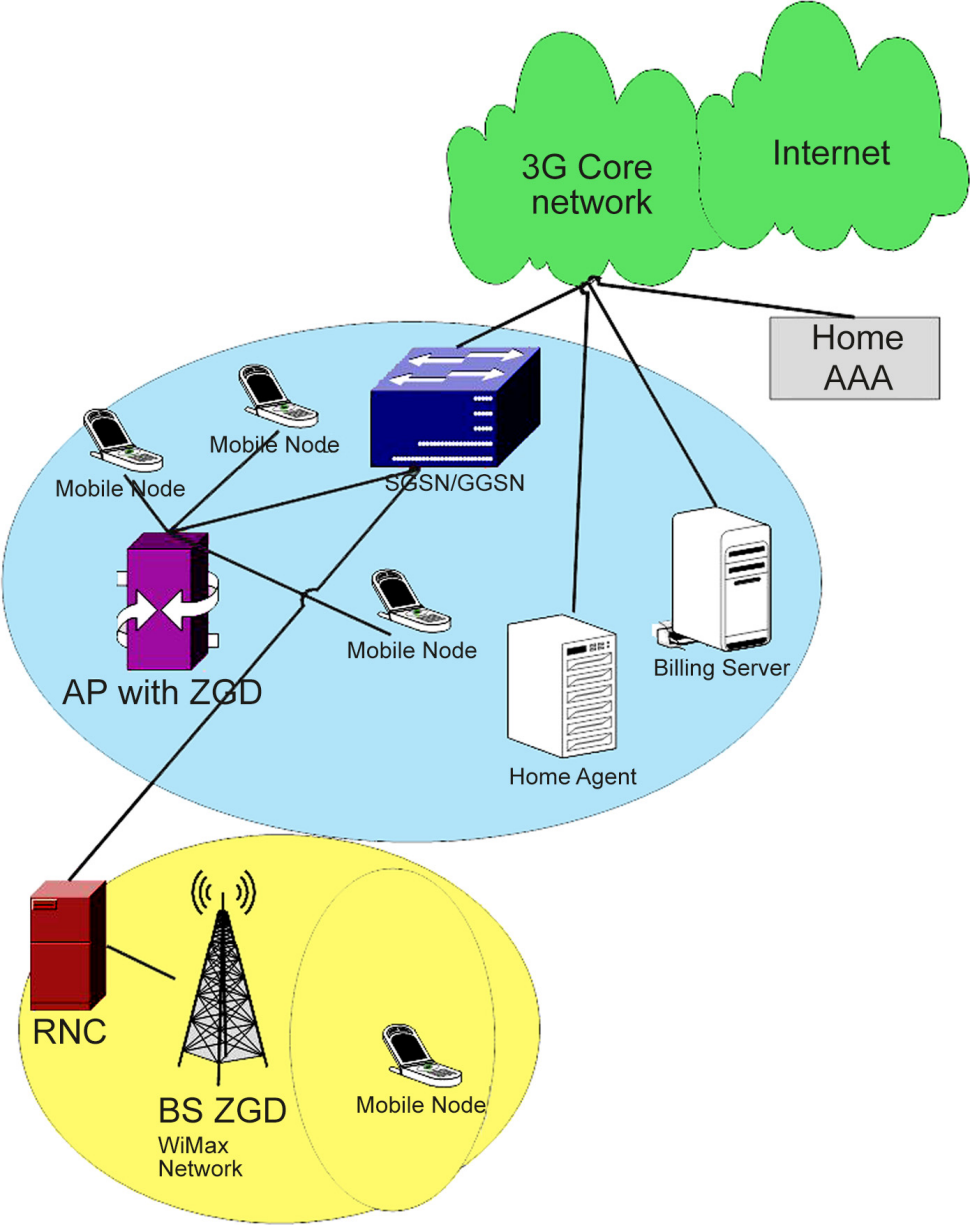
Architecture of proposed system. Heterogeneous environment with zone gateway decision head.

The system assumes that the mobile terminal is able to access any network and the built-in interfaces of the network. "Fig 1" presents the architecture of a heterogeneous environment with a zone gateway decision head incorporated into the access point of network. The infrastructure consists of a serving GPRS support node and Gateway GPRS support node. The SGSN through radio network control (RNC)/Base switching Control (BSC) is responsible for mobility management. Integration with such elements as the Home Subscribe Server/Home Location Server to manage services and provide external access to IP networks is also implemented in SGSN. The GGSN, which is the IP access point to the internet for the mobile terminal, has a virtual private network or another access network. A WiMax network contain critical components for subscription and traffic control from the base station within an access network. The mobile nodes obtain information on the access network through the controller of the network. The AAA server authenticates, authorizes and provides accounting functions. Periodic updates are conducted, and the collective information is sent to the nearby access network for the decision-making process. When the network detects that the signal strength on the mobile node is weak during roaming, the zone gateway decides to initiate handover; moreover, this gateway provides the access information on the foreign network through the controller. The information provides the QoS of the other network, and the mobility management protocols enable the mobile to connect to the best network through of the operation of the proposed mechanism.

### 3.2 QoS Requirements for Handover Decision Scheme

Allocation of network resources becomes a major challenge in providing QoS during vertical handover. The QoS schemes should reduce the overhead and additional complexity for the traffic characteristics. In order to enable application with different QoS requirement, the key components for user experience and choice to switch with suitable network capability, the traffic characteristics are classified first as conventional for VoIP, video conference application, second streaming for audio and video streaming application, third interactive for web browsing, gaming messages and finally background traffic like file transfer and e-mail type applications. QoS will have impact by specific policies adopted dynamically and the network decision to facilitate handover by new session set up through ranking the network by Multiple Criteria Decision Making method brings optimal policy. The QoS parameters considered for traffic classes are RTT, network reliability, traffic handling priority, signal strength etc with the corresponding weight for traffic class computed using the AHP technique. However, management of vertical handover in a heterogeneous network requires proper selection of various metrics. Selection of network parameters for vertical handover have great impact on signaling overheads. First stage of the proposed work takes signal strength and network reliability to provide solution to unnecessary handover computations. Traffic handling priority parameter enables network congestion check and offer load balance. The cost factor is also added for choice of the network along with RTT to have reduced connection failure. To achieve effective system performance in terms of throughput available bandwidth is considered in dynamic programming approach to avoid ping pong effect. Each parameter is specified by a weight that indicates its relative importance. The Analytical Hierarchical Processing (AHP) aids in determining the weights. AHP determines the alternative measures from known scales. The process brings clear picture of all the essential alternatives that influence the outcome of a decision. The rating for the criteria with decision alternatives are scaled form 1 to 9, where 1–3 denotes the 'less' preferred criteria score and 4–6 are assigned 'medium' score for the parameter and 7–9 range denotes the high score for the alternatives The different QoS requirements for four classes of traffic are shown in "Table 1" and are computed to derive the weights for a mobile node.

**Table 1:** Decision Metrics for Various Traffic Classes.

Criteria	Traffic Class 1	Traffic Class 2	Traffic Class 3	Traffic Class 4
Signal strength	9	9	8	9
Traffic handling priority	6	3	7	3
RTT	6	7	3	7
Reliability	9	2	9	2
Cost	1	6	7	6

### 3.3 Knapsack Problem Formulation

The knapsack problem is a multi-constrained integer programming model. From a theoretical point of view, knapsack applications are not only limited to packing problems but also cargo loading, cutting stocks and budget control. This motivates application of dynamic programming approach to the problem of deriving optimal solutions for network selection in a heterogeneous network. The problem formation considers the loading of n mobile nodes into the candidate network. Each mobile node is assigned a weight w_i_ according to the QoS traffic classes and the score value obtained from the TOPSIS model. The proposed vertical handover decision is integrated together with TOPSIS and is incorporated into the server of the access point/base stations of the wireless network to finalize the target network resource availability based on the collective information obtained from the nearby access point optimal solution. The network is divided into zones to remove centralized decisions. This zoning concept amends the priority order of the access network, and handovers are initiated based on the traffic of the available network. Moreover, the proposed approach allows the zone gateway to make handover decisions on behalf of mobile nodes. Hence, signaling on the wireless portion of the network is reduced, which reduces signaling delay with the impact of a change in a vertical handover scheme and thereby improves the delay performance. The handover decision is initiated on detection, i.e., when the point of attachment load exceeds a predefined threshold and variations in radio link are originated at the mobile terminal. However, due to the limited information available at the mobile terminal, the proposed approach makes every effort to provide the best network association to the mobile terminals with QoS guarantees. The optimal decision directs the mobile users using the handover initiation procedure as presented in the algorithm. Computation is performed for n number of mobile nodes, which requires handover to be performed and connected to the candidate access network W with total capacity. Access point V_i_ accepts a certain number of mobile nodes in the handover process. Hence, the objective is to maximize the resource utilization of the access point such that,

$$\text{Max }\sum_{i=0}^{n}{\text{V}}_{\text{i}}$$ (1)

subject to

$$\sum b_{i}\leq W$$ (2)

An array v[0….n, 0….B] is constructed for 1 ≤ i ≤ n, and 1 ≤ b ≤ B. The access point V[i,b] stores the maximum of n mobile nodes {1,2……….i}. Computation of mobile nodes with an array V[n,b] allows maximum network utilization, and hence, the solution to the problem is obtained. The optimal solution is found recursively with initialization v[0,b] = 0 when the candidate network is not selected for *1 ≤ b ≤ B* and v[i,b] = -∝ for b <0.

To compute v[i,b], v[i-1] becomes the storage limit for the mobile node i. The remaining mobile nodes are v[i-1,b-b_i_] if 0 ≤ b ≤ w.

Thus,

$$\text{V}\left[\text{i},\text{B}\right]=\{\begin{array}{c} \text{max}(\text{v}\left[\text{i}-1,\text{b}\right],{\text{v}}_{\text{i}}+\text{v}\left[\text{i}-1,\text{b}-{\text{b}}_{\text{i}}\right])\\ 0 \end{array}$$ (3)

When array temp [i,b] becomes 1, then the decision to take mobile node i to v[i,b] is performed. If temp [n,b] is 1, then the network has reached the maximum capacity and handoff is not performed. The steps are repeated for temp [n-1,b-b_n_]. Thus, an optimization approach for construction of the decision process for the stage i is formed. A feasible decision depends on the current condition of the stage. There exists a finite number of stages in the decision process. Dynamic programming observes the principles of optimality that facilitates application to any complex problems. Through these approach, the partial solution can be optimally extended to sequences of decision operation. However, the article limits the storage range from V[i,b] to V[i-1] with network utilization for mobile nodes. Hence, infeasible amount of memory space computation may seem to be complex occasionally. Still dynamic programming is most efficient on well designed VHD and such performance are illustrated in simulation results. The transition is governed by Eq (3) from one state to other with decision variables w_i_, w_i-1_,w_i-2_,—-, w_0_, and the optimization problem becomes

$${\text{V}}_{\text{i}}{\text{(s}}_{\text{i}}{\text{) = Max [F}}_{\text{i}}{\text{(w}}_{\text{i}}{\text{, s}}_{\text{i}}{\text{)+ f}}_{\text{i-1}}{\text{(w}}_{i\text{-1}}{\text{, s}}_{i-1}{\text{) + --- + f}}_{\text{0}}{\text{(w}}_{\text{0}}{\text{, s}}_{\text{0}}\text{)]}$$ (4)

subject to,

*S*_*m– 1*_ = *t*_*m*_(W_m_,S_m_) for m = 1 to n

*w*_*n*_ ∈ *W*_*m*_ for m = 0 to n

where

w_i_ denotes the decision parameter chosen from decision set w_i_

s_i_ denotes the state at the i^th^ stage of the process, for i = 1 to n.

**Algorithm**

I ← *Mobile nodes i = 0 to n connected to ZGD*

B← *threshold value of bandwidth*.

b← *bandwidth occupied by access network*

v_i_← *point of attachments*

W← *candidate access network*

V[i,B]← *access point with maximum load*

K← *alternative access network selected*

**Procedure**

Knapsack(v,b,n,B)

{

For (b = 0 to B)V[0,b] = 0;

For (i = 1 to n)

 For (b = 0 to W)

IF((b(i)<b) and (v[i] +V [i—1, b-b[i]] > V [i -1,b]))

  {

V[i,b] = v[i] +V[i-1, b-b[i]];

Temp[i,b] = 1; // handoff avoided

  }

  Else

  {

V[i,b] = V[i-1,b];

Temp[i,b] = 0;

  }

 K = W  //perform handoff

For (i = n down to 1)

IF(W[i,K] = = 1

 {

 SELECT i; // Mobile i selected for handoff

 K = K-b[i];

 }

RETURN V[n,W

### 3.4 TOPSIS for Vertical Handover

Technique In Order To Preference by Similarity to Ideal Solution (TOPSIS) is one of the MADM approaches used to rank alternatives. This approach provides the rankings of the best networks through parameter evaluation. To reduce unnecessary handover computation, the rank score of TOPSIS alone is insufficient to improve the QoS requirement. The network finds the best optimal solution that has a critical impact on the network capacity. The TOPSIS method selects the alternative that is the closest to the ideal solution and farthest from the most negative alternative. The following steps describe the TOPSIS approach.

**Step 1**: Assumption of m alternatives and n attributes/criteria and assignment of the score for each option with respect to each criterion. Let x_ij_ be the score of option i with respect to the criterion j.

**Step 2**: Construction of normalized decision matrix that transforms the various attribute dimensions into non-dimensional attributes, thus allowing comparisons across criteria.

The normalized scores or data are

$${\text{for r}}_{\text{ij}}\text{=}\frac{{\text{x}}_{\text{ij}}}{\left(\sum {\text{x}}_{\text{ij}}^{2}\right)}\text{ for i = 1, }\ldots \text{, m; j = 1, }\ldots \text{,n }$$ (5)

**Step 3**: Construction of the weighted normalized decision matrix that takes a set of weights for each criteria w_j_ for j = 1,…n.

**Step 4**: Multiplication of each column of the normalized decision matrix by its associated weight.

**Step 5**: Formation of the new matrix with elements of

$$v_{ij}=w_{j}r_{ij}$$ (6)

**Step 6**: Determination of ideal and negative ideal solutions

The ideal solution is

$${\text{A}}^{*}\;=\;\left\{{\text{ V}}_{1}{}^{*},\;\ldots ,{\text{ V}}_{\text{n}}{}^{*}\right\}$$ (7)

where

$${\text{V}}_{\text{j}}{}^{*}\;=\;\{\text{ max }({\text{V}}_{\text{ij}})\text{ if j }\epsilon \text{ J; min }({\text{V}}_{\text{ij}})\text{if j }\epsilon \text{J'}\}$$

and the negative ideal solution is

$$\text{A'}=\;\left\{{\text{ V}}_{1}{}^{\text{'}},\;\ldots ,{\text{ V}}_{\text{n}}{}^{\text{'}}\right\},$$ (8)

where

$${\text{V' = \{min (V}}_{\text{ij}}\text{) if j }\in {\text{ J; max (V}}_{\text{ij}}\text{) if j }\in \text{ J'}\}$$

**Step 7**: Calculation of the separation measures for each alternative.

The separation from the ideal alternative is

$${\text{S}}_{\text{i}}^{*}\;={\left[\sum {{\text{(V}}_{\text{j}}^{*}{\text{-V}}_{\text{ij}})}^{2}\right]}^{1/2}\text{i = 1, }\ldots \text{ m}$$ (9)

Similarly, the separation from the negative ideal alternative is:

$${\text{S}}_{\text{i}}^{\text{'}}\;={\left[\sum {{\text{(V}}_{\text{j}}^{\prime }{\text{-V}}_{\text{ij}})}^{2}\right]}^{1/2}\text{i = 1, }\ldots \text{ m}$$ (10)

**Step 8**: Calculation of relative closeness to the ideal solution Ci*

$${\text{C}}_{\text{i}}^{*}\;={\text{S}}_{\text{i}}^{\text{'}}\;/\;\left({\text{ S}}_{\text{i}}^{*}+{\text{S}}_{\text{i}}^{\text{'}}\;\right)0<{\text{C}}_{\text{i}}^{*}<1$$ (11)

The option with Ci* closest to 1 is selected.

## Simulation Result

Simulations are performed to evaluate the performance of the proposed method. The reference scheme adopted here for performance is between WiFi network to WiMax technology. WiMax adopts combination of modulation schemes such as Quadrature Phase shifting keying and Quadrature Amplitude Modulation and the multiplexing schemes OFDM (Orthogonal Frequency Division Multiplexing) to enable high range access and high throughput performance. WiMAX release 2.0 also known as IEEE 802.16m is the IEEE candidate for the 4th generation of telecommunication networks and its goal is set to meet all the specifications of IMT-Advanced. Also literature by Bessie Malila et al [20] brings performance evaluation of a next generation network through WiMax network. The paper reveals that WiMax is one of the technologies designed to meet the demands of the application and the results obtained show that the network conforms to ITU-T performance standards and can deliver integrated NGN services. Literature also showed work carried for vertical handover adopted through WiMax by Nadine Akkari et al [21]. The paper proposed inter-domain management module for guiding the vertical handover to WiMax network. Literature by Mohamed K Watfa et al [22] revealed that WiMax is designed to be an powerful network service provider for next generation network using IPv6. As WiMax solution stand out from the competition by decreasing the deployment costs and increasing the network revenue, the simulation scheme is illustrated with WiMax. The proposed scheme can also be supported by 3G and LTE systems. Integration architecture models such as loose coupling and tight coupling enable roaming between 3GPP and non 3GPP systems. Simulation tool used is ns2.29 network simulator. The performance measure with WiMax and WiFi network are considered for illustration. Two IEEE 802.16 base station and two WiFi access point are configured with variable number of mobile nodes. The total simulation time is 200 s and CBR traffic of 500, 1000, 1500, and 2000Kbps was created between nodes. At the start MN are connected to point of attachment to WiFi network and on mobility the mobile nodes move towards WiMax network. The topology adopted is flat grid. The performance velocity is varied from 10Km/h to 20 Km/h with random mobility for mobile nodes. In order to establish quick route set up on triggering of VHD, AODV routing protocol is used. AODV is one of the best integrated multicast routing protocol. Since the proposed work is network controlled and the optimal solution on vertical handover has to be initiated to mobile nodes. The reliability of AODV makes limited periodic updates of mobile node for mobility management Also this routing protocol affords the method to less overhead, guarantee loop freedom in paths. Hence the article presents the performance measure of delay, throughput and handover computations through AODV routing protocol. The simulation is repeated number of times and average value is taken to increase the accuracy of observed result. The simulation parameters are presented in "Table 2". The proposed decision mechanism operates together with the access point/base station of the wireless network to manage the access to the network. The first test evaluates the handover scenario between the WiMax and Wi-Fi network for two velocity cases of 10 km/h and 20 km/h.

**Table 2:** Simulation Metrics.

Topography	1000*1000
No. of Nodes	25
No. of PoA/BS	4
Routing protocol	AODV
Wireless standard	802.16, 802.11
Packet size	500-2000Kbps

### (a) Handover Computation Result

**Fig 2:**
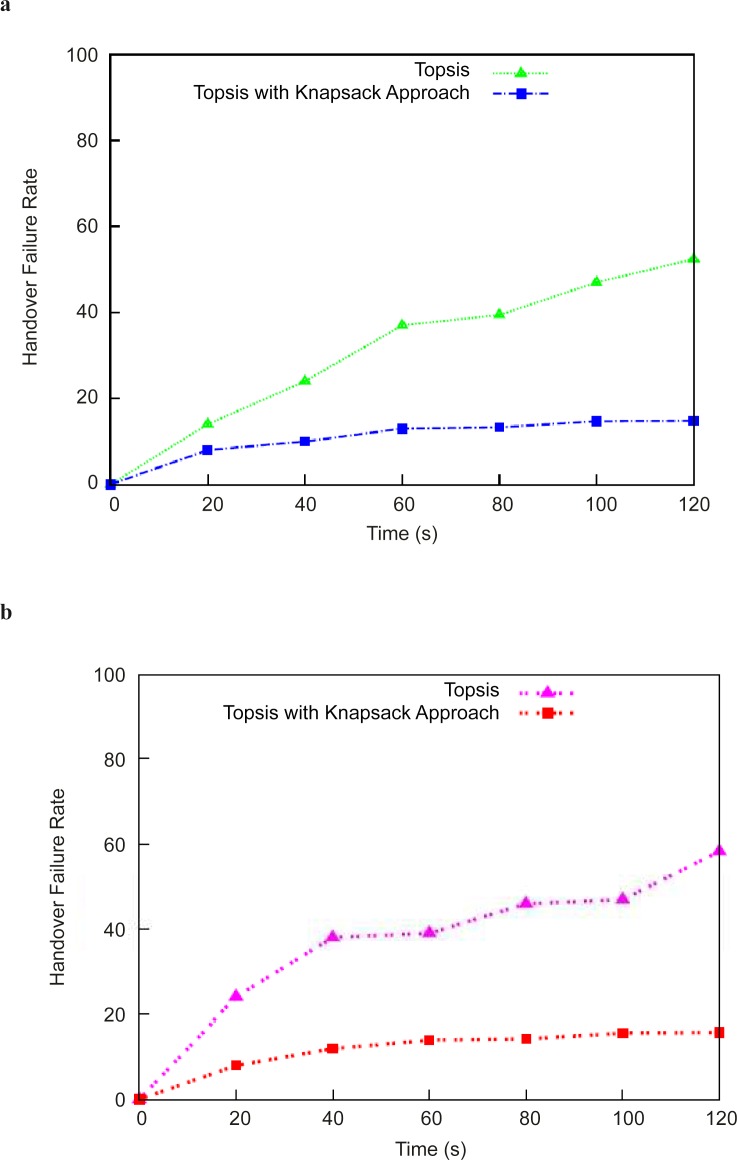
Handover failure rate computation(a) Results with velocity 10 Km/h (b) Results with velocity 20 Km/h.

The comparative results for handover failure are shown in "Fig 2" with the TOPSIS method and the TOPSIS method with Knapsack approach with the two velocity scenarios. This paper compares the result of the proposed method with existing TOPSIS method presented in literature [6] [23]. The PoA1 and PoA2 denote the access points of the Wi-Fi, and PoA3 and PoA4 denote the base stations of the WiMax network. The QoS selection criteria are measured, and the handover decisions are performed by the optimization method at the correct time to avoid unnecessary handover between the two different networks. The mobile nodes are assisted by the optimal knapsack problem formulation for handover initiation. The TOPSIS method in the computation illustrates that a handover event from PoA1 to PoA3 occurred multiple times, but the proposed method initiated handover via the correct decision and attached the node to the other base station of the network; thus, an optimal handover decision was made to reduce an unnecessary handover event. This result shows that the resources of the network can be utilized effectively compared with the existing observation.

**Fig 3:**
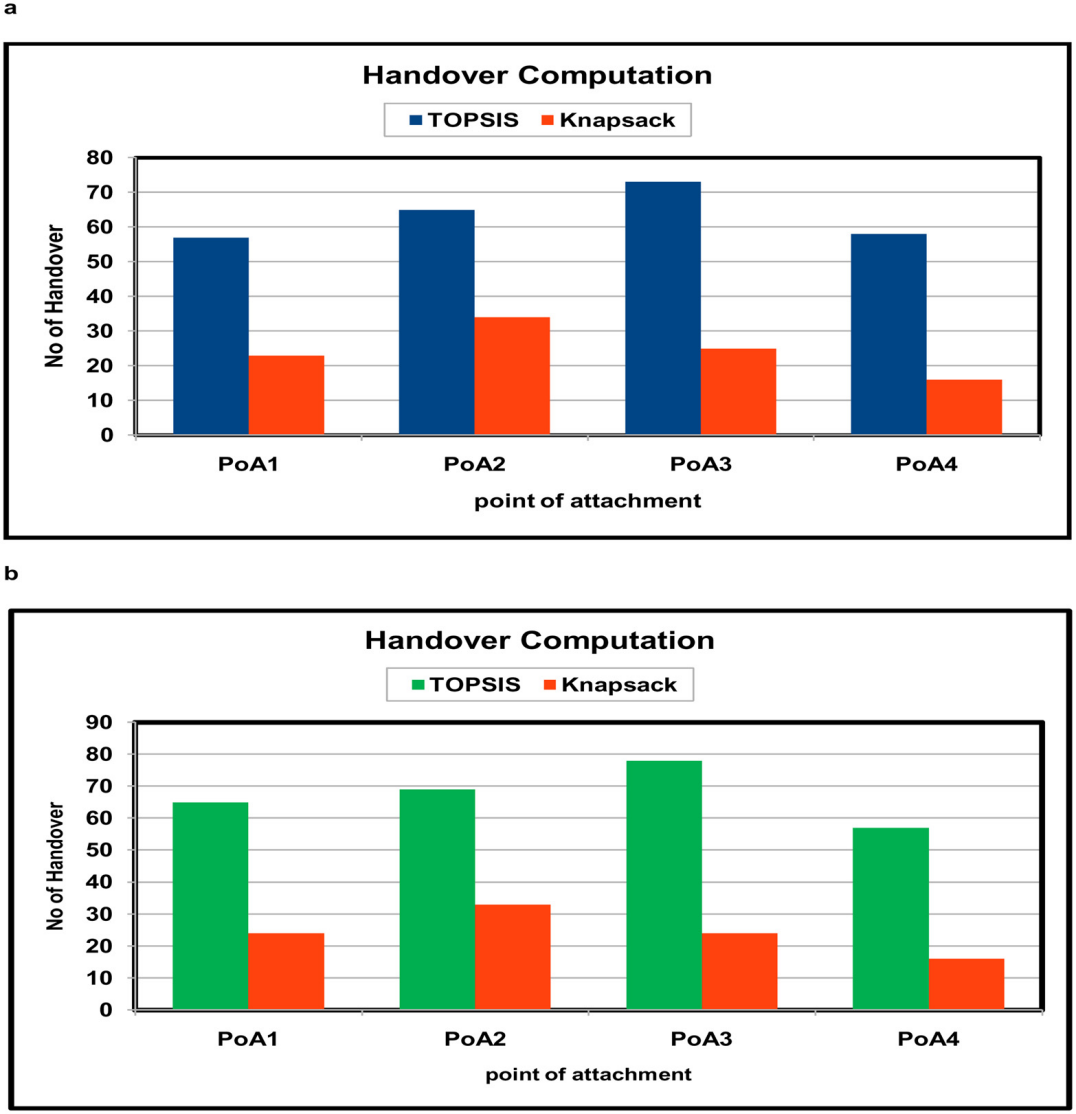
Total Handover Computation between WIMAX and WI-FI network(a) velocity 10 Km/h (b) velocity 20 Km/h.

The result was also tested in the same simulation environment with a mobile velocity of 20 km/h. "Fig 3" shows the total number of handovers performed during the simulation time, and the proposed method avoids unnecessary handover events. When the TOPSIS approach makes the decision to switch the mobile node from PoA1 to PoA3, the handover was immediately turned back to PoA1 because the load at PoA4 could not accommodate the extra traffic. Using the optimal decision, the mobile node moved with greater speed, the connection to PoA5 was optimal, and thus, the traffic signaling in the network was reduced.

### (b) Throughput Measurement Analysis

**Fig 4:**
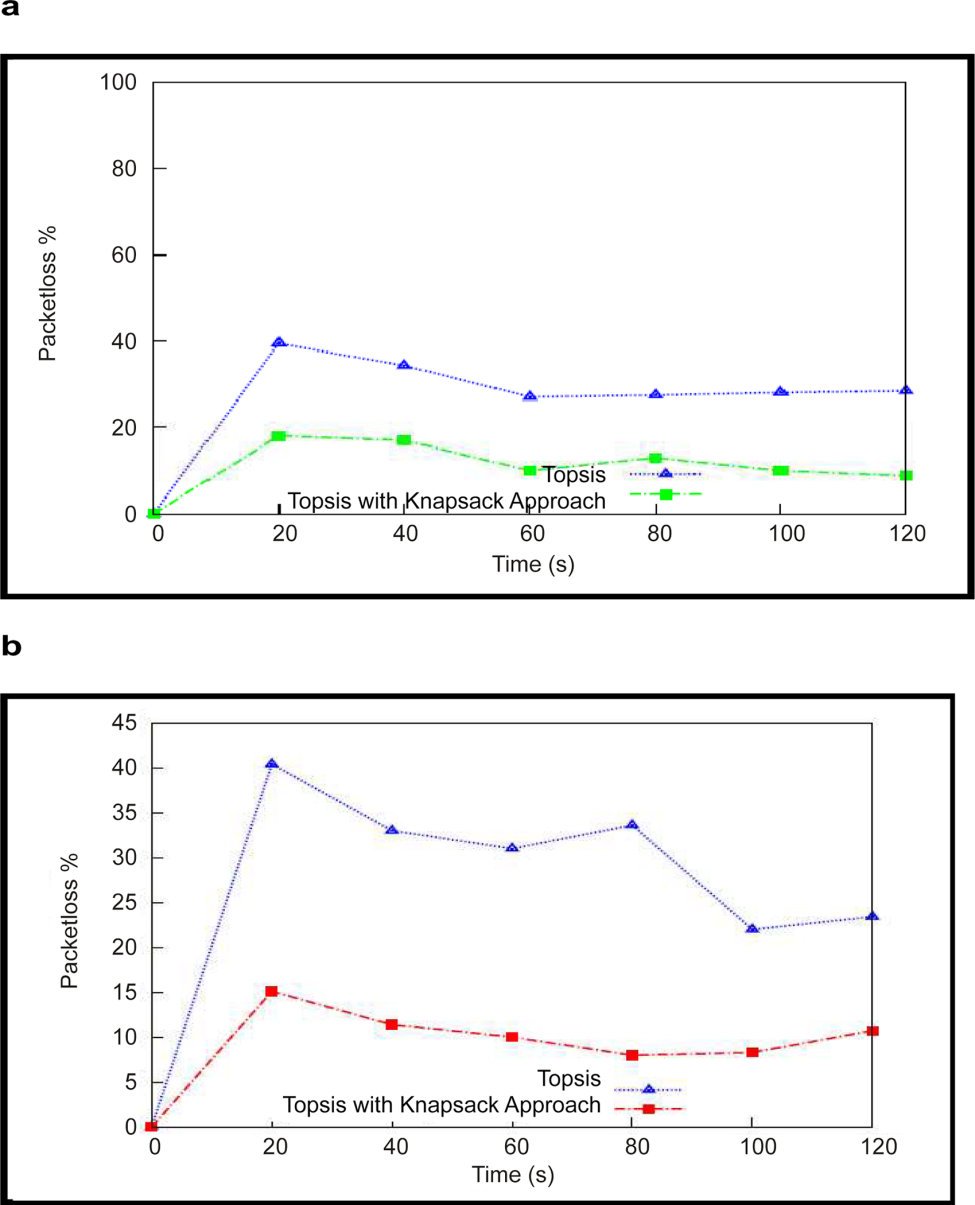
Packet transfer computation (a)Results with mobile velocity 10 Km/hmobile (b)Results with velocity 20 km/h.

The second component of the work tested for packet loss during the vertical handover process. Throughput is measured by the amount of data transmitted by the mobile node after a set of matching decisions during the defined period. The total number of packets transmitted by the source node and the number of packets received by the destination node are recorded to calculate the throughput. The results showed that the packet drop was considerably reduced in the network. "Fig 4" shows the throughput result against the TOPSIS method. The packet loss due to multiple handover decisions affects the network performance in TOPSIS. However, by introducing the Knapsack approach into the network, the access point enables the best handover decision on behalf of the mobile node and accurately identifies the bandwidth available for the mobile node. The same simulation setup was used for a velocity of 20 km/h, and the performance is improved compared with the TOPSIS method.

### (c) Delay Measurement Analysis

**Fig 5:**
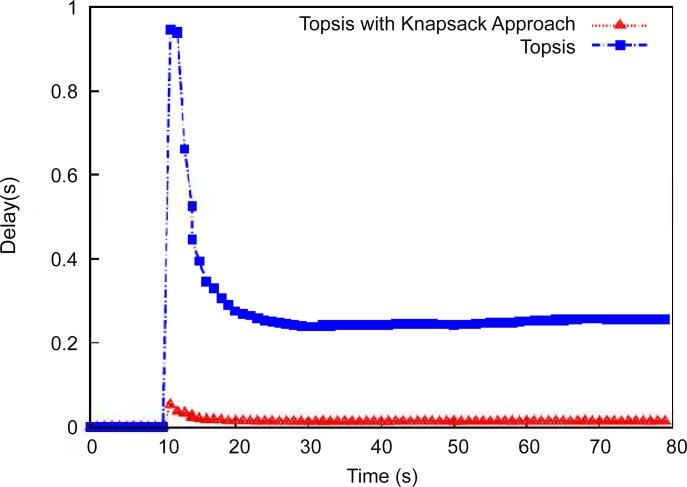
Delay performance between WIMAX and WiFi network with 10 km/h.

The third section of our study measures the end-to-end delay during handover initiation. The best handover decisions aid the mobile node to utilize network resources effectively and allow better QoS support. **"** Fig 5" shows the delay performance obtained by the proposed mechanism with a velocity of 10 km/h. The network initiates the handover at the correct time, and the network controller knows to which network the mobile node should be connected. The collective ranking provides the best network selection to the mobile nodes, and QoS selection enables the mobile user to more effectively use the services of network.

## Conclusion

The vertical handover mechanism is vital for fast switchover from one network to another in a heterogeneous wireless network. A wide range of network characteristics motivates implementation of a multi-criteria-based handover decision algorithm together with dynamic programming of the Knapsack model. This article presents a QoS-aware handover decision algorithm with the QoS parameters of signal strength, RTT, traffic handling priority, reliability and cost for different traffic classes. Simulation with NS2 is performed between WiMax and Wi-Fi networks with two different velocity scenarios. The proposed scheme can also be supported by 3G and LTE systems. In case of LTE reference system integration architecture models such as loose coupling and tight coupling enable roaming between 3GPP and non 3GPP systems. Assumptions are considered for the mobility patterns and communication between pairs of access points and base stations. The result obtained by implementing the proposed mechanism demonstrates that unnecessary handover is reduced by 24% more compared with that existing TOPSIS model, and packet throughput is increased to 12% more compared with existing systems [6]. The system also showed that the result for end-to-end delay was reduced on a larger scale compared with the system performance using intelligent handover algorithms. The TOPSIS with Knapsack vertical handover decision approach proved to work better than TOPSIS alone for handover decisions by reducing the complexity in implementation.

## Current Limitations and Future Research Direction

There are few open issues that need to be further investigated for vertical decision making process. The examples include security support during handovers through authentication schemes. Fact authentication schemes enable handover decision to distribute the context to the target candidate network which may improve the performance during handover process. Authentication schemes are designed in literature [24],[25] to reduce the challenges of security issues in next generation network and obtain reduced cost benefit and delay performance during vertical handover. These literatures revealed that authentication based algorithms reduce delay to nearly 50ms. However, the proposed article did not focus nor implemented security related issues and in future direction of research, this great novel ideal shall be implemented for NGN. Also the reference scheme adopted for simulation is WiMax network. This work can be extended to LTE system. Cell-edge soft handover can be dropped from the LTE system, hence coupling architecture may help LTE to increase the performance. Integration architecture models such as loose coupling and tight coupling are introduced between 3GPP and non 3GPP systems. In future, this proposed work can be implemented along with coupling architecture to measure the performance results.

## Supporting Information

S1 Data Set (XLSX)
